# Long-Term Cultivation of Human Atrial Myocardium

**DOI:** 10.3389/fphys.2022.839139

**Published:** 2022-02-23

**Authors:** Maximilian J. Klumm, Christian Heim, Dominik J. Fiegle, Michael Weyand, Tilmann Volk, Thomas Seidel

**Affiliations:** ^1^Institute of Cellular and Molecular Physiology, Friedrich-Alexander-Universität Erlangen-Nürnberg, Erlangen, Germany; ^2^Department of Cardiac Surgery, Friedrich-Alexander-Universität Erlangen-Nürnberg, Erlangen, Germany

**Keywords:** human atrium, calcium imaging, tissue culture, *in vitro*, confocal microscopy, gene expression, refractory period

## Abstract

Organotypic culture of human ventricular myocardium is emerging in basic and translational cardiac research. However, few institutions have access to human ventricular tissue, whereas atrial tissue is more commonly available and important for studying atrial physiology. This study presents a method for long-term cultivation of beating human atrial myocardium. After written informed consent, tissues from the right-atrial appendage were obtained from patients with sinus rhythm undergoing open heart surgery with cardiopulmonary bypass. Trabeculae (pectinate muscles) prepared from the samples were installed into cultivation chambers at 37°C with a diastolic preload of 500 μN. After 2 days with 0.5 Hz pacing, stimulation frequency was set to 1 Hz. Contractile force was monitored continuously. Beta-adrenergic response, refractory period (RP) and maximum captured frequency (f_max_) were assessed periodically. After cultivation, viability and electromechanical function were investigated, as well as the expression of several genes important for intracellular Ca^2+^ cycling and electrophysiology. Tissue microstructure was analyzed by confocal microscopy. We cultivated 19 constantly beating trabeculae from 8 patient samples for 12 days and 4 trabeculae from 3 specimen for 21 days. Functional parameters were compared directly after installation (0 d) with those after 12 d in culture. Contraction force was 384 ± 69 μN at 0 d and 255 ± 90 μN at 12 d (*p* = 0.8, *n* = 22), RP 480 ± 97 ms and 408 ± 78 ms (*p* = 0.3, *n* = 9), f_max_ 3.0 ± 0.5 Hz and 3.8 ± 0.5 Hz (*p* = 0.18, *n* = 9), respectively. Application of 100 nM isoprenaline to 11 trabeculae at 7 d increased contraction force from 168 ± 35 μN to 361 ± 60 μN (*p* < 0.01), f_max_ from 6.4 ± 0.6 Hz to 8.5 ± 0.4 Hz (*p* < 0.01) and lowered RP from 319 ± 22 ms to 223 ± 15 ms. CACNA1c (L-type Ca^2+^ channel subunit) and GJA1 (connexin-43) mRNA expressions were not significantly altered at 12 d vs 0 d, while ATP2A (SERCA) and KCNJ4 (Kir2.3) were downregulated, and KCNJ2 (Kir2.1) was upregulated. Simultaneous Ca^2+^ imaging and force recording showed preserved excitation-contraction coupling in cultivated trabeculae. Confocal microscopy indicated preserved cardiomyocyte structure, unaltered amounts of extracellular matrix and gap junctions. MTT assays confirmed viability at 12 d. We established a workflow that allows for stable cultivation and functional analysis of beating human atrial myocardium for up to 3 weeks. This method may lead to novel insights into the physiology and pathophysiology of human atrial myocardium.

## Introduction

Organotypic long-term culture of adult human and animal cardiac tissue is emerging as a promising tool in basic and translational cardiac research (Fischer et al., 2019; Ou et al., 2019; Qiao et al., 2019; Watson et al., 2019; Perbellini and Thum, 2020). By preserving the multicellular structure and function of intact myocardium for up to several weeks *in vitro*, the technique bridges the gap between cell culture experiments on the one hand and *in vivo* studies using laboratory animals on the other. Myocardial tissue culture prevents the quick degradation and dedifferentiation occurring during culture of isolated adult cardiomyocytes (Banyasz et al., 2008), but still provides a level of control over physical and chemical stimuli that cannot be achieved *in vivo*. Another important advantage is that the technique can be used to cultivate fully differentiated human myocardial tissue obtained from explanted hearts or accrued during open heart surgery. Thus, the method provides an experimental platform for basic scientists to investigate, for example, the long-term effects of drugs on human cardiac tissue (Fischer et al., 2019; Miller et al., 2020). Other applications include the establishment of structure-function relationships in diseased human myocardium (Abu-Khousa et al., 2020), the investigation of viral myocardial infections (Bojkova et al., 2020), or the maturation of stem cell-derived human cardiomyocytes (Lu et al., 2021). Because stem-cell derived engineered heart tissues have not yet reached the differentiation, multicellularity and physiological properties of adult myocardium, the “heart in a dish” approach of myocardial tissue culture can currently be considered the most realistic *in vitro* model.

Earlier approaches to myocardial slices [reviewed in de Boer et al. (2009)] were useful mainly for short-term experiments (Han et al., 2002; Bussek et al., 2009) or used non-adult heart tissue (Bursac et al., 2003; Pillekamp et al., 2005). Mostly, however, tissues were not functionally stable for more than 24–48 h. Then, one study reported that ventricular slice cultivation on top of cell culture filters prolonged myocardial function and viability, possibly a result of preload applied through adhesion forces together with improved oxygen supply at the liquid-air interface (Brandenburger et al., 2012). However, the structure and function of the tissues still degraded over the course of several days, and for assessment of contractility slices had to be transferred from the filters to baths containing force transducers. This hampered repeated functional measurements over the cultivation period. In subsequent studies it was found that, in addition to a suitable culture medium, there are three important requirements for the longevity of human and animal ventricular slices: (1) the application of diastolic preload or stretch to obtain a physiological resting sarcomere length, (2) regular electrical stimulation causing contraction, and (3) convection of the culture medium (Fischer et al., 2019; Watson et al., 2019). Both studies used M199 as culture medium and carbon electrodes for electrical field stimulation. Watson et al. (2019) used a caliper to determine the strain necessary to achieve an optimal sarcomere length of approximately 2.2 μm and perfused the culture chamber with oxygen-enriched medium. Fischer et al. (2019) applied a preload of 0.66 mN/mm^2^ and agitated the medium by placing culture chambers on a rocking platform. Other studies suggested similar solutions for electrical stimulation, preload, and convection or oxygen enrichment of the medium (Ou et al., 2019; Qiao et al., 2019).

Techniques for the cultivation of human ventricular slices are increasingly used in basic and translational studies addressing mechanisms of heart failure, a syndrome resulting mainly from ventricular dysfunction. However, if the described cultivation techniques could be applied also to human atrial tissue, this would constitute an *in vitro* model of comparable utility for studying atrial physiology and pathomechanisms, most prominently of atrial fibrillation. Similarly as described for ventricular slice culture, this could address the need for multicellular three-dimensional *in vitro* models of atrial myocardium (van Gorp et al., 2020) and complement stem-cell derived chamber-specific models (Zhao et al., 2019). Furthermore, because human atrial tissue can be easily obtained during open heart surgery with cardiopulmonary bypass, it is available to a larger number of laboratories than human ventricular tissue. Therefore, we asked whether it is possible to cultivate human atrial myocardium as reported for ventricular slices.

This study presents and evaluates a method for the long-term cultivation of human right-atrial trabeculae using the method reported by Fischer et al. (2019). It describes the preparation, culture conditions and several methods for the assessment of important functional parameters, such as contractile performance and kinetics, Ca^2+^ signaling, as well as gene expression and tissue microstructure before and after cultivation.

## Materials and Equipment

Specifications of buffers, media, chemicals, reagents, antibodies, dyes, PCR primers, devices, and tools are provided in the Supplementary Material.

## Methods

### Tissue Collection

Collection and use of patient tissue was approved by the ethics committee of the University of Erlangen-Nürnberg. All experiments followed the Declaration of Helsinki ethical principles. Tissues were donated after written informed consent by patients undergoing open heart surgery with cardiopulmonary bypass at the Cardiac Surgery Department of the University Hospital Erlangen, Germany. Patients with a history of atrial fibrillation were excluded from this study. Patient characteristics are presented in Table 1. Before insertion of a two-stage venous cannula for partial cardiopulmonary bypass, the tip of the right-atrial appendage (RAA) was resected after setting up purse-string sutures. The resected tissue, which would have been discarded otherwise, was immediately placed in cool (4°C) storage solution and transported to the laboratory on ice within 20 min.

**TABLE 1:** Characteristics of patients who donated samples of the right atrial appendage.

Patient characteristics	
*N*	11
Age	67.1 ± 12.5 years
Male	10 (91%)
BMI	28.1 ± 6.1 kg/m^2^
Sinus rhythm	11 (100%)
Surgery type *CABG* *Aortic valve*	9 (82%) 2 (18%)
Hypertension	11 (100%)
Hypercholesterolemia	8 (72%)
Diabetes mellitus	4 (36%)
Coronary heart disease	10 (91%)
Myocardial infarction	4 (36%)
LVEF	55.6 ± 7.0%

*BMI, body mass index; CABG, coronary artery bypass graft; LVEF, left-ventricular ejection fraction.*

A video showing the tissue collection during surgery is available in the Supplementary Material.

### Preparation of Trabeculae for Tissue Culture

**FIGURE 1:**
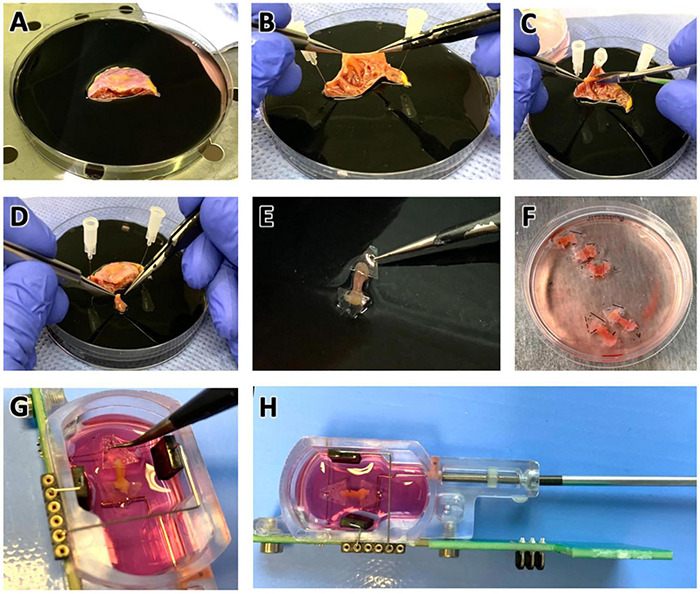
Preparation of pectinate muscles (trabeculae) from right atrial appendage (RAA) samples for tissue culture. **(A)** RAA obtained during cardiac surgery, stored and transported in cold cardioplegic solution. Diameter of the petri dish is 100 mm. **(B)** Inspection of the RAA interior, displaying the typical trabecular meshwork. **(C)** Trabeculae were carefully dissected with a scalpel while fixing the tissue with pins on a rubber layer. **(D)** Excised trabecula. **(E)** Plastic triangles glued to the longitudinal ends of the trabecula. **(F)** Prepared trabeculae in cold Tyrode’s solution containing BDM in a standard tissue culture petri dish. Diameter of the displayed dish is 60 mm. **(G)** Trabeculae were installed into cultivation chambers by mounting the plastic triangles on a spring wire (top) and a stiff wire (bottom). **(H)** Adjustment for trabecula length and setting of a defined preload is achieved by moving the stiff wire, which is attached to a bolt and nut, using a hex screwdriver. See also the Supplementary Videos 1, 2.

An overview of the tissue processing steps is shown in Figure 1. The tissue was transferred to a 100 mm petri dish containing cold (4°C) storage solution with 30 mmol/l butanedione monoxime (BDM) and a sterile rubber pad for ease of processing (Figure 1A). BDM, an uncoupling agent, is used in many slicing protocols (e.g., Watson et al., 2017), and preparations of cardiac muscle strips (Mulieri et al., 1989) to reduce tissue damage. BDM is also used to improve cardiac and skeletal muscle function after long cold-storage periods (Stringham et al., 1994; van der Heijden et al., 2000) and to reduce reperfusion injury (Voigtlander et al., 1999). All processing steps were performed on a cooled plate on a laminar flow workbench. After a lateral incision the tissue was unfolded and secured with sterile cannulas pinned into the rubber pad (Figure 1B). Individual trabeculae were dissected using forceps, micro-scissors and, if necessary, magnifying glasses (Figure 1C). Typically, four to six trabeculae were retrieved from one single specimen (Figure 1D). For gene expression analysis one trabecula was snap-frozen in liquid nitrogen and stored at −80°C. The remaining trabeculae were prepared for installation into cultivation chambers of a commercially available cultivation system (InVitroSys, Germany) as previously described (Fischer et al., 2019). For this, trapezoid polyether holders with holes for mounting were glued to both endings of the trabecula (Figures 1E,F) with a 2-butyl cyanoacrylate tissue adhesive (Surgibond, SMI). Subsequently, the trabeculae were installed in the culture chambers filled with prewarmed (37°C) culture medium by mounting the plastic holders with forceps on two opposing metal wire posts inside the chambers (Figure 1G). The chambers were immediately placed on the cultivation platform in the incubator. A diastolic load of 500 μN was applied by retracting one of the wires manually with a hex key (Figure 1H). Up to 8 chambers were incubated in parallel. The incubator provided a constant temperature of 37°C, 5% CO_2_ and 80–85% relative humidity. The rocker for medium agitation was set to 60 rpm, and electrical field stimulation was provided by carbon electrodes with 50 mA constant current and a 3 ms biphasic pulse. A biphasic pulse regiment (3 ms negative pulse, 1 ms pause, 3 ms positive pulse) is important to prevent electrochemical imbalance during long periods of electrical field stimulation. Otherwise, we found that electrolysis of the culture medium may occur, producing toxic chemicals and significant changes in pH. Stimulation frequency was set to 0.5 Hz for the first 48 h of culture. Twitch forces of trabeculae were continuously measured and recorded using a magnetic sensor mechanism and a flexible spring wire as described previously (Fischer et al., 2019). Stimulation time points were recorded in synchrony with the contraction data. After an adaptation period of 48 h the pacing frequency was set to 1 Hz.

A video showing all steps of trabecula preparation as well as a step-by-step protocol are available in the Supplementary Material.

### Maintenance of Tissue Culture

Culture medium was partly exchanged with prewarmed fresh medium every 24–48 h. To compensate for evaporation, 0.8 ml were removed and 0.9 ml added every 24 h or 1.6 ml removed and 1.8 ml added every 48 h. After the first 24 h of culture, the preload was readjusted to 500 μN to account for viscoelasticity and plasticity of the tissue. On days two, seven and twelve, the beta-adrenergic response was investigated by adding 100 nM isoprenaline to the chambers. Directly before and 5 min after addition of isoprenaline, pacing protocols were performed. After 30–45 min, isoprenaline was washed out by completely replacing the medium. Except for four trabeculae from three patient samples, all trabeculae were removed from culture after 12 days. Trabeculae were then either fixed for immunofluorescence staining, frozen at −80°C for gene expression analyses, or used to assess viability or intracellular Ca^2 +^ transients.

### Analysis of Force Recordings

**FIGURE 2:**
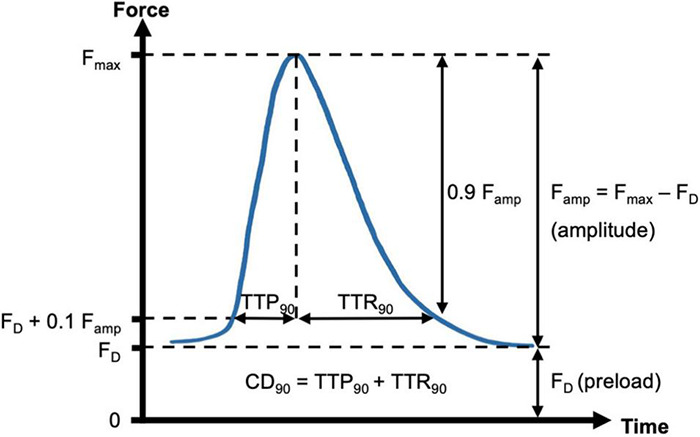
Schematic of contraction analysis. Diastolic force (F_D_, preload) and maximum force (F_max_) were respectively determined from the minimum and maximum spring wire deflections during a contraction cycle, multiplied by the spring constant. The force of contraction was then identified with the resulting amplitude, F_amp_ = F_max_ - F_D_. Contraction kinetics were assessed by using F_D_ + 10% of F_amp_ as a threshold for start and end of a contraction. Thus, the parameters contraction duration (CD_90_), time to peak (TTP_90_) and time to relaxation (TTR_90_) were calculated.

Force of contraction and contraction kinetics were analyzed as previously described (Abu-Khousa et al., 2020). Figure 2 shows one contraction cycle with the diastolic force F_D_, which is set by the preload and stretches the trabecula. After an electrical stimulus, the trabecula begins to contract, which deflects the flexible spring wire in the cultivation chamber. The deflection is registered by a magnetic field sensor and used to calculate the corresponding force with the spring constant of approximately 25 mN/mm. As threshold for the beginning of a contraction, 10% of the amplitude was used. At this point, the contraction duration was calculated (CD90). The time from 10% to the maximum force was defined as the time to peak force (TTP90). The time from the maximum back to 10% of the force was defined as time to relaxation (TTR90). Thus, CD90 equals TTP90 + TTR90. These parameters were determined computationally, using custom-written Matlab functions (Mathworks, Matlab versions 2019a and higher). After downsampling the recorded data from 400 to 200 Hz with a mean filter, a central moving median filter of radius 10 and, subsequently, a central moving mean filter of radius 5 were applied to reduce noise. Only periods with the agitation rocker stopped were analyzed. First, the noise level was estimated by calculating the standard deviation and maximum amplitude at low pacing frequencies (≤1 Hz) during 200 ms intervals before an electrical stimulus. Contraction durations were always shorter than 500 ms. Thus, during the 200 ms intervals directly before a stimulus, the fluctuations in the recorded sensor data stem exclusively from noise. Next, a peak detection was performed with the minimum peak height set to the maximum noise amplitude plus one standard deviation of the noise. Starting from each peak (F_max_) a time window of ± 50% of the stimulus interval duration was analyzed to obtain the contraction amplitude (F_amp_ = F_max_ − F_D_). F_D_ was defined as the minimum force within the interval and then subtracted from the signal. Moving backward in time from the peak, TTP90 was then obtained by identifying the time of first occurrence of a force ≤ 0.1 F_amp_ before the peak. Conversely, TTR90 was obtained by identifying the time of first occurrence of force ≤ 0.1 F_amp_ after the peak. CD90 was calculated as the sum of TTP90 and TTR90. The calculated parameters of all beats within an analyzed period were averaged and then considered representative for this period. For example, if the rocker was stopped for 2 s at a pacing frequency of 3 Hz, six contraction cycles were analyzed and the resulting mean parameters were representative for 3 Hz. The correct detection and analysis of beats was verified by inspection of the analyzed intervals and detected peaks and by comparison of the number of electrical stimuli with the number of detected beats. This way, it was also possible to decide whether a pacing frequency was captured.

### Pacing Protocols to Determine Refractory Period and Maximum Frequency

Within 15 min of starting tissue culture (day 0) and on days 2, 7 and 12, pre-defined pacing protocols were performed to assess the refractory period (RP) and maximum captured frequency (*f*_max_). For this purpose, the trabeculae were paced with a baseline frequency of 0.5 Hz. After 22 s of normal pacing, three intertwined S2 beats followed with a defined S2 beat interval. After another 22 s of regular pacing at 0.5 Hz, trabeculae were stimulated with the frequency corresponding to the S2 beat interval for 6 s. For example, if the S2 beat interval was 500 ms, the corresponding pacing frequency was 2 Hz. This scheme was repeated with decreasing S2 beat intervals and accordingly increasing frequencies. The S2 beat intervals were (in ms): 750, 500, 400, 300, 275, 250, 240, 230, 220, 210, 200, 190, 180, 170, 160, 150, 140, 130, 120, 110, and 100. To evaluate contraction parameters at frequencies other than the baseline pacing rate, the stimulation frequency was increased successively from 0.2 to 5 Hz as follows (in Hz): 0.2, 0.5, 0.75, 1.0, 1.5, 2.0, 2.5, 3, 3.5, 4.0, 4.5, and 5.0. Each period lasted for 25 beats, for example, 25 s at 1 Hz and 5 s at 5 Hz. During Ca^2 +^ imaging, modified versions of these protocols with fewer steps were used to reduce protocol durations. During all pacing protocols, the rocker of the culture platform was stopped intermittently for 2–10 s to record at least six consecutive contractions at the end of each stimulation period without any artifacts from medium agitation. These artifacts are caused by movements of the spring wire resulting from inertia and gravity and appear as low-frequency noise in the recorded data (see Figure 5B for an example). Thus, stopping the rocker briefly improves the signal-to-noise ratio. It was shown in previous work that stopping medium agitation has no effects for approx. 20–30 s (Fischer et al., 2019). Only contractions recorded during rocking-free periods were analyzed. The stimulation schedules of the protocols were defined with commands in a text file, which were read by the control software and then submitted to the controller/stimulation unit. The controller transferred a 400 Hz data stream including the force transducer data and the electrical stimulation times.

Contractions during the pacing protocols were identified by noise and peak analysis as described above. To decide whether a S2 beat was captured, the interval between the S2 stimulus and the following S1 stimulus was analyzed. If a peak, i.e., any positive slope above the minimum peak height, was detected, the stimulus was considered as captured. A second criterion was that all three S2 beats had to be captured. During the decreasing S2 stimulus intervals, the interval before the first occurrence of “not captured” was identified with the refractory period. To determine the maximum captured frequency, the last five stimuli of the high-frequency pacing train were analyzed applying the described approach for peak detection. If any of these five stimuli was not captured, the frequency was considered as not captured. The last captured frequency before the first occurrence of a non-captured frequency was identified with *f*_max_. By reviewing the contraction traces and detected peaks manually by researchers, it was ensured that the analysis program behaved as expected.

### Parallel Registration of Contractile Force and Intracellular Ca^2+^

Cultured trabeculae were mounted in a modified MyoDish culture chamber (InVitroSys) with a glass cover slip bottom. Culture medium was exchanged for Ca^2+^ imaging buffer. Calbryte 520-AM Ca^2+^ indicator (AAT Bioquest) was added at 10 μmol/l together with 0.1% Pluronic Acid F127 (Biotium) and incubated for 20 min at 37°C and agitation on a horizontal laboratory shaker. The chamber was then installed on an inverted microscope (Leica, DMIRB). For dye washout superfusion with Ca^2+^ imaging buffer (37°C) was initiated at 250 ml/h and maintained throughout the experiment. After 5 min the trabeculae were stimulated with pacing protocols for analysis of the force-frequency relationship (FFR), RP, and *f*_max_, using the MyoDish control unit. Simultaneously the trabecula was illuminated with a spectrum of 460–500 nm through a 5× lens (Leica, 0.15 HC PL FLUOTAR). Emitted light was collected after bandpass filtering 510–585 nm with a photomultiplier of a fluorescence imaging system (IonOptix). Contraction and Ca^2+^ signals were synchronized using the electrical simulation signal recorded with both acquisition systems.

### Immunofluorescent Staining

Tissue was fixed either directly after procurement or after 12 days in culture with paraformaldehyde (PFA, 2% in phosphate buffered saline, PBS) for 30 min. After two washing steps in PBS (performed after all following steps) the specimens were embedded in Tissue Tek O.C.T. Compound (*Sakura*, 4583), frozen at −20°C and cut into 80 μm sections in longitudinal orientation. Subsequently, the tissue sections were stained with primary antibodies against connexin-43 (Cx43, 1:400) and alpha-actinin (aACT, 1:200) in PBS supplemented with 1% bovine serum albumin, 5% normal-goat-serum and 0.25% TritonX-100 for 24–48 h. Secondary antibodies goat-anti-rabbit AF555 (used for Cx43, 1:400) and goat-anti-mouse AF488 (used for alpha-actinin, 1:200) were incubated for 24–48 h while protected from light. In a last step, nuclear staining with DAPI and staining of the extracellular matrix with wheat germ agglutinin (WGA) conjugated to AF647 was performed for 8–12 h. Stained tissue sections were then mounted on standard microscope slides in Fluoromount G mounting medium, covered with a coverslip and dried at room temperature for at least 24h while protected from light.

### Confocal Microscopy and Image Analysis

Confocal microscopy and investigation of tissue microstructure was performed according to previously published methods (Seidel et al., 2016, 2017a,2017b; Abu-Khousa et al., 2020). Microscopy slides were mounted on the stage of an inverted confocal microscope (Zeiss, LSM780) and imaged with a 63× oil objective (Zeiss, Plan-Apochromat, 63×/1.4). Three-dimensional image stacks (1280 × 1280 × 100 voxels, voxel size 0.1 μm × 0.1 μm × 0.2 μm) of three randomly selected regions were scanned from each sample. After image acquisition, raw data were noise-filtered and deconvolved with the Richardson-Lucy method, using measured point spread functions, and corrected for depth-dependent signal attenuation (Seidel et al., 2016). Afterwards, the WGA, aACT, Cx43 and DAPI signals were separated from background by application of intensity thresholds calculated for each image stack. Global thresholds of image mode plus 1, 3, and 2 standard deviations were used for WGA, Cx43, and DAPI, respectively. The aACT signal showed large variation in intensity across image stacks. Therefore, a local threshold was used. This was achieved by application of a box mean filter with a dimension of 20 × 20 × 2 voxels and subsequent subtraction of the result from the original image. A threshold of mode + 1 standard deviation was then applied to the resulting image.

The amount of extracellular matrix was identified with the volume ratio of the segmented WGA signal (Emde et al., 2014; Seidel et al., 2017b). Similarly, gap junction density was estimated from the volume density of the Cx43 signal after application of a three-dimensional morphological opening filter with a radius of 0.2 μm to reduce speckle noise. Sarcomere density was estimated from the volume density of the segmented aACT signal. The aACT was also used as a myocyte marker to calculate the myocyte volume fraction. After watershed-based creation of intracellular and extracellular segments on the WGA signal inverted distance map (Seidel et al., 2013), those segments with an aACT content of at least 7.5% were classified as myocyte segments.

### Viability Assay

Cellular viability of atrial tissue was assessed with MTT assay as described in earlier studies (Brandenburger et al., 2012). In brief, fresh or cultured trabeculae were washed once with assay buffer and then incubated in 2 ml assay buffer supplemented with 0.5 mg/ml 3-(4,5/dimethyl-2-thiayolyl)-2,5-diphenyl-2H-tetrazoliumbromide (MTT, Sigma) on the cultivation platform inside the incubator for 20 min, i.e., in the dark at 37°C, 5% CO_2_ and 80–85% relative humidity. After that, the tissue was washed twice with assay buffer and dry-frozen at −80°C. For photometric analysis, reduced formazan was extracted from tissue by incubation in 1 ml dimethylsulfoxide (DMSO) at 37°C for 30 min and constant agitation at room temperature. The absorbance of the resulting supernatant was measured against DMSO in a microplate reader (Tecan, GENios) at 595 nm excitation. For normalization, total protein of the tissue was determined by lysis of the trabeculae after formazan extraction in 100 μl sodium hydroxide solution (0.5 M) for 30 min at 95°C. Lysed samples were then used for Bradford protein quantification assay. For this, samples were diluted 1:10 in Bradford reagent (BioRad) and measured against buffer in a microplate reader at 595 nm excitation. For absolute protein quantification a serial dilution of bovine serum albumin samples was used followed by linear regression for the calculation of total protein content of the samples. The formazan absorbance was then divided by the total protein, yielding the relative absorbance as an indicator of tissue viability.

### Gene Expression Analysis

Quantitative real-time PCR (qPCR) was performed using total RNA extracted from frozen tissue samples that were stored at −80°C using NucleoSpin RNA-Kit (Macherey-Nagel), following the manufacturers’ instructions. Tissue was processed with a T10 basic homogenizer (IKA) in the lysis buffer of the kit prior to all following steps. RNA was eluted in ultrapure water, quantified and quality controlled by assessing A260/280 nm and A260/230nm ratios with a DS 11 + spectrophotometer (Denovix). 50 ng of total RNA was transcribed to cDNA with QuantiTect RT-Kit (Qiagen), including a DNAase digestion step for genomic DNA removal. The resulting cDNA was separated via gel electrophoresis to exclude genomic DNA contaminants and cDNA degradation. For real-time quantification, 50 ng cDNA was used in SYBR Select assay (Thermo Fisher Scientific) in a StepOnePlus thermocycler (Applied Biosystems) using the following protocol: Initiation: 50°C for 120 s, 95°C for 120 s, Amplification (40 cycles): 95°C for 15 s, 60°C for 60 s. The genes of interest and the reference genes were amplified with pairs of oligonucleotides as primers. Cycle threshold (C_t_) values of triplicate reactions were averaged for each sample and non-template controls ensured the absence of contaminating DNA. Reaction efficiencies were calculated for every experiment from linear regression analysis of a template dilution serious (1:5, 1:25, 1:125, and 1:625). C_t_ value differences (ΔC_t_) between treatment groups and the control group (fresh) were calculated and corrected for reaction efficiencies. Relative expression differences (ratios) were determined by normalization to values of the reference genes EEF2 and HPRT1, which were calculated accordingly (Pfaffl, 2001).

### Statistics

If not otherwise indicated, data are presented as mean ± standard error of the mean (SEM). The paired, two-sided *t*-test was used to compare parameters obtained from the same trabecula, the Welch’s (unequal variance), two-sided *t*-test was used to compare parameters from different trabeculae. Where required due to multiple comparisons of one parameter, the Holm-Bonferroni method was used to correct *p*-values. The level of significance (alpha) was set to 0.05.

## Results

### Viability of Cultured Human Atrial Trabeculae

**FIGURE 3:**
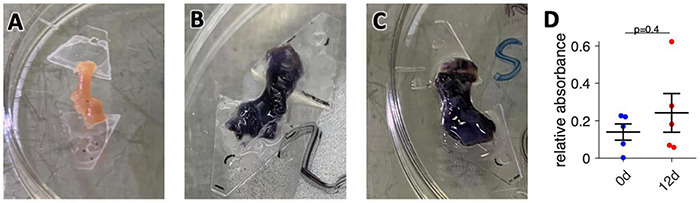
Assessment of tissue viability. **(A)** Photograph of an unstained trabecula. Width of the plastic triangles is 7 mm. **(B)** Photograph of a fresh trabecula after incubation with the tetrazolium dye MTT for 15 min. The intensity of the dark purple color indicates the amount of MTT enzymatically reduced to formazan. **(C)** Photograph of a trabecula cultivated for 12 days after 15 min of MTT incubation from the same specimen as panel **(B)**. **(D)** Results from photometric analysis of the amount of formazan produced from MTT, normalized to total protein of fresh trabeculae (0 d) and trabeculae from matching specimens cultivated for 12 days (12 d). *N* = 5/5 trabeculae/samples. The unequal variances *t*-test was applied for statistical analysis.

We prepared a total number of 38 trabeculae from 11 specimens obtained from 11 patients (Table 1). Of these, 33 (87%) produced contractions exceeding the sensor noise (>50 μN) in response to electrical stimulation immediately after bringing them into culture. The five non-beating trabeculae were removed. Five beating trabeculae were used within 30 min for viability assays, leaving 28 trabeculae. If after 2 days no response could be detected even after addition of isoprenaline, the trabeculae were removed and not included in any data analysis, which was the case in 5 trabeculae. Thus, after 12 d in culture, 23 trabeculae (82% of 28) were still beating. Of these, four trabeculae from three different specimens were kept in culture for a total of 21–22 days, while the other trabeculae were used at day 12 for functional, structural and molecular analyses. To assess general viability, we used an MTT assay, which indicates the activity of cellular NAD(P)H-dependent oxidoreductases reducing the nearly colorless tetrazolium dye MTT to the purple formazan (Figure 3). Thus, the amount of formazan produced is related to the metabolic activity and viability of the cells present in the tissue (Berridge et al., 2005). When comparing 5 trabeculae at 0 d with 5 trabeculae from matching specimens after 12 d in culture, we found no significant difference in relative absorbance, i.e., formazan produced in relation to total protein content was comparable (Figure 3D). This indicates that tissue viability of beating trabeculae was preserved during long-term culture.

### Contractile Force During Culture

**FIGURE 4:**
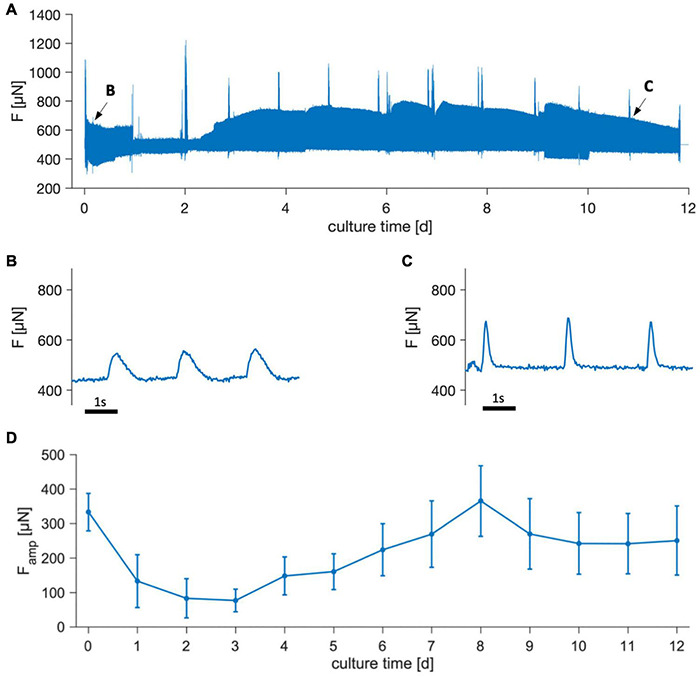
Contraction force of trabeculae cultivated for 12 days. **(A)** Overview of an example showing the measured force, *F*. Spikes result from pacing protocols, addition of isoprenaline and medium exchanges. **(B,C)** Magnifications of periods indicated by arrows in panel **(A)**, showing single contractions. Pacing rate during the displayed periods was 0.5 Hz. Rocking for medium agitation was turned off, causing the amplitudes to appear smaller than at the corresponding time points in panel **(A)**. **(D)** Mean contraction force amplitudes, *F*_amp_, of 22 trabeculae from 8 tissue samples. Error bars indicate SEM.

Figure 4 shows the contraction force of trabeculae cultivated for 12 days. In the example shown (Figures 4A–C) it is visible that contractility was relatively high directly after starting tissue culture, but then declined during the subsequent days. In some trabeculae, contraction was not detectable between days 1 and 3, but recovered to initial values within 1 week. Note that the trace shown in Figure 4A includes artifacts from tissue agitation, which add to the actively developed force. This explains why the beats presented in Figures 4B,C appear to have lower amplitudes than visible in the overview because the rocker was stopped. Spikes visible in the overview were caused due to transiently increased contractility after medium exchange, likely caused by brief changes in medium pH, and also due to periodically performed stimulation protocols or beta-adrenergic stimulation on days 2, 7, and 12. In Figure 4D the mean contraction amplitude of 22 trabeculae is presented over 12 days, showing a similar trend as in the example. Initial contraction force was reached after approximately 7 d and was then stable for the remaining time. Four trabeculae were kept in culture for 3 weeks, and contraction amplitudes did not significantly differ from day 12. From this we conclude that during the first days of culture contractility is reduced, but recovers within 1 week and then remains stable for at least the next 2 weeks.

### Assessment of Refractory Period and Maximum Captured Frequency

**FIGURE 5:**
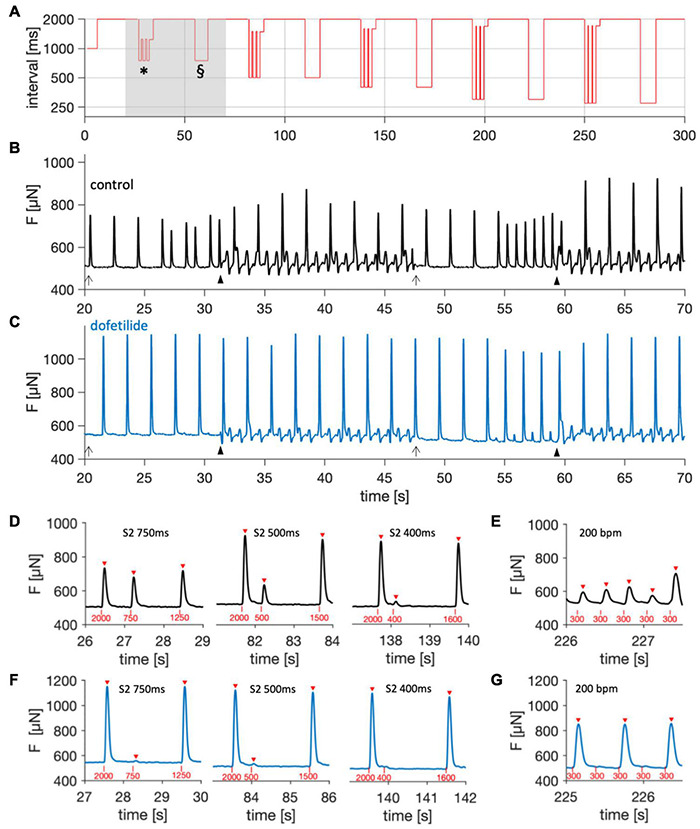
Assessment of refractory period (RP) and maximum frequency captured (*f*_max_). **(A)** Stimulation intervals during the pacing protocol, showing successive periods of decreasing interval lengths intermitted by 20-s periods with a baseline interval of 2000 ms. After each S1-S2 beat period (*), a period with constant pacing at the corresponding frequency (§) followed. Note the logarithmic *Y*-axis scale. **(B,C)** Force, *F*, measured during the period marked by the gray area in panel **(A)** of a trabecula **(B)** before, and **(C)** 15 min after treatment with 100 nM dofetilide. Closed arrow heads indicate the start of medium agitation by the rocker, causing shaking artifacts. Open arrow heads indicate rocker stop. **(D)** S1-S2 beat periods before addition of dofetilide (control) at S2 beat intervals of 750, 500, and 400 ms. Red triangles indicate detected contractions, red vertical bars and numbers indicate stimulation time points and intervals, respectively. **(E)** Period of pacing with 3.3 Hz (200 bpm) before addition of dofetilide (control). **(F)** S1-S2 beat periods after addition of dofetilide at S2 beat intervals of 750, 500, and 400 ms (same trabecula as **D,E**). **(G)** Period of pacing with 3.3 Hz (200 bpm) after addition of dofetilide.

Figure 5 displays an example of the pacing protocol used to assess RP and *f*_max_. The stimulation times were used to create the graph shown in Figure 5A, where the stimulus-to-stimulus intervals are displayed over time. It is visible on the logarithmic scale that the S2 beat intervals decreased with each period, and that each S2 beat period was followed by a brief period of stimulation with the corresponding frequency. Figure 5B shows the registered contraction data of an S2 beat period and the subsequent high-frequency stimulation intermitted by 22 s of baseline pacing at 0.5 Hz. The example also demonstrates how stopping the rocker reduced artifacts from medium agitation. To verify the relation of refractory period to action potential duration, we applied the highly selective IKr blocker dofetilide, which significantly prolongs the atrial action potential duration (APD) and refractory period (Gwilt et al., 1991; Sedgwick et al., 1992; Tsujimae et al., 2007). Figure 5C depicts the response to exactly the same stimulation sequence as displayed in Figure 5B, 15 min after addition of 100 nM dofetilide to the culture medium. At close inspection one can appreciate an increase in contractile force, presumably caused by increased Ca^2+^ influx due to APD prolongation, as well as a diminished response to the S2 stimulus. Furthermore, the high-frequency period (55–58 s) shows alternans, with each beat of high amplitude followed by a beat of markedly reduced amplitude. The effect of dofetilide becomes more evident when comparing the S2 beats under control conditions (Figure 5D) with those after application of the drug (Figure 5F). While the S2 beat at an interval of 750 ms was nearly unchanged when compared with the baseline beats under control conditions, it was hardly visible with dofetilide. According to the defined criteria, the refractory period in the shown example was 400 ms (control) and 500 ms (dofetilide), that is, dofetilide increased RP by approx. 25%, fitting well to clinical data (Gwilt et al., 1991). As expected, *f*_max_ was decreased after dofetilide application (Figures 5E vs. G). In the example it decreased from 5 to 2.5 Hz. These results demonstrate that RP and *f*_max_ as assessed by the chosen pacing protocols and criteria may indirectly detect changes in action potential duration.

### Effects of Culture on Contraction Parameters, Refractory Period, and Maximum Captured Frequency

**FIGURE 6:**
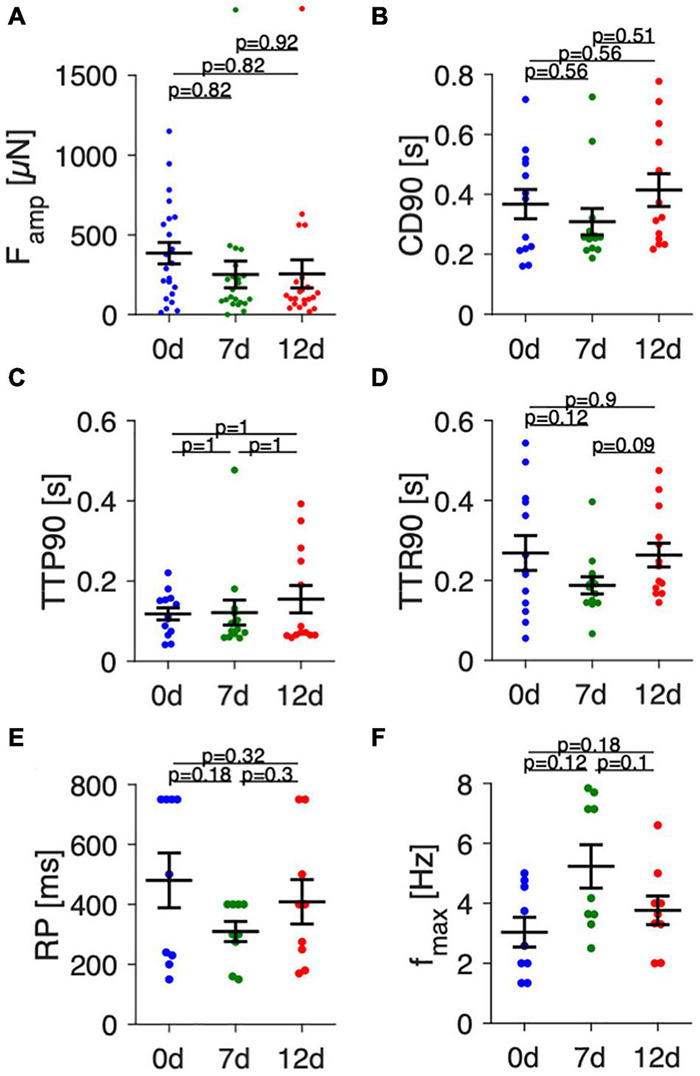
Analysis of contraction amplitudes, kinetics and pacing response in fresh and cultivated trabeculae. **(A)** Contraction force amplitudes (*F*_amp_) of fresh (0 d) trabeculae and trabeculae cultivated for 7 (7 d) and 12 days (12 d) at 1 Hz pacing. **(B)** Contraction duration (CD90), **(C)** time to peak (TTP90), and **(D)** time to relaxation (TTR90) at 1 Hz pacing. **(E)** Refractory period (RP), **(F)** Maximum frequency captured (*f*_max_). *(N)* = 22/7 trabeculae/samples in panel **(A)**, 13/7 in panels **(B–D)**, and 9/6 in panels **(E,F)**. A paired, two-sided *t*-test with Holm-Bonferroni multiple-comparison correction was applied.

Figure 6 presents statistical data of contraction parameters, RP and *f*_max_ obtained from 22 trabeculae. Figure 6A shows that initial contraction forces at 0 d showed high variability, with values ranging from nearly 0 to 1200 μN. After 1 week of culture, variability was smaller, but the mean did not change significantly. This was also true after 12 d in culture. Analysis of contraction kinetics (Figures 6B–D) was restricted to trabeculae with amplitudes larger than 50 μN. Thus, the parameters of some trabeculae could not be evaluated at all time points, reducing the number of matching data points to 13. Mean CD90 was 367 ± 51 ms at 0 d, 309 ± 45 ms at 7 d and 414 ± 57 ms at 12 d, but differences were not statistically significant. Similarly, TTP90 and TTR90 did not differ at the investigated time points. Mean values ranged from 118—155 ms and 188–263 ms, respectively. However, with a contribution of approximately 35% to CD90, TTP90 was significantly shorter than TTR90 (*p* < 0.0001, paired *t*-test), fitting well to the observation that atrial contraction is about twofold faster than relaxation (Palka et al., 1996). RP showed high variance, especially at 0 d (Figure 6E) and did not change significantly over time. However, due to the chosen pacing protocol, the resolution was lower at long S2 beat intervals with relatively large steps from 750 to 500 ms and then to 400 ms. This may explain the large deviation of RP from the mean found in some trabeculae. Analysis of *f*_*max*_ (Figure 6F) revealed a tendency toward higher values at 7 d (5.2 ± 0.8 Hz) vs 0 d (3.0 ± 0.5 Hz) and 12 d (3.8 ± 0.5 Hz) without reaching statistical significance. Collectively, the data suggest that the variance between different trabecula is relatively high, but that culture of up to 12 d does not lead to significant changes in contraction parameters, RP or *f*_max_.

### Simultaneous Recording of Ca^2+^ Signals and Contraction

**FIGURE 7:**
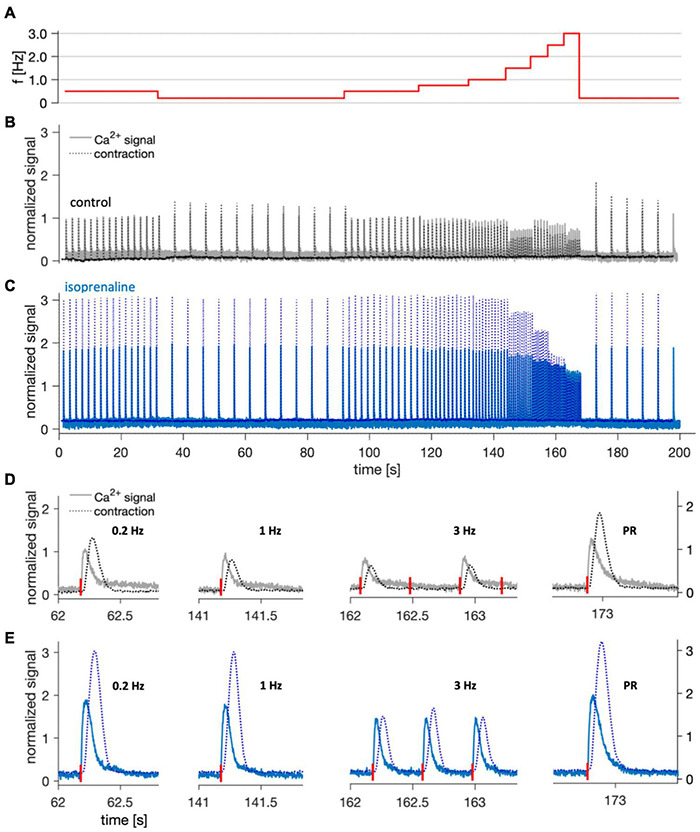
Example of simultaneous Ca^2+^ signal and contraction recording at 37°C in a trabecula after 22 days in culture. **(A)** Stimulation frequencies applied during the imaging to assess the force-frequency response and post-rest beat. **(B)** Overlay of normalized Ca^2+^ signal (solid line) and contraction force (dotted line) recorded during the shown stimulation protocol under control conditions. After offset subtraction, both signals were normalized to the respective amplitude in response to the first stimulus (0.5 Hz). **(C)** The same trabecula 5 min after application of 100 nM isoprenaline. **(D,E)** Normalized single contractions and corresponding Ca^2+^ signals recorded at different stimulation frequencies, and the post-rest (PR) beat at 5 s after the last 3 Hz stimulus. Stimulation times are indicated by red vertical bars next to the *X*-axis. Shown traces are magnifications of panels **(B,C)**, respectively.

Because Ca^2+^ cycling in atrial cardiomyocytes is gaining increasing interest and is altered in atrial fibrillation (Hove-Madsen et al., 2004; Denham et al., 2018), we intended to demonstrate that cultivated trabeculae can be used for simultaneous recording of intracellular Ca^2+^ and contraction (Figure 7). After loading a trabecula that had been in culture for 3 weeks with the Ca^2+^ indicator Calbryte, we placed the cultivation chamber on a microscope equipped with a perfusion system and a fitting excitation light source and emission filters. Using a 5× lens and a photomultiplier to record a sum signal of the whole trabecula and by capturing and recording the stimulation voltage pulses, we were able to synchronize the Ca^2+^ signal with the recorded force data. Starting from a baseline pacing frequency of 0.5 Hz, a stimulation sequence of increasing frequency was applied to investigate the behavior of contractility and the Ca^2+^ signal at frequencies up to 3 Hz (Figure 7A). Each frequency was held for 12 consecutive stimulation pulses. After the last 3 Hz stimulus, the pacing was decreased to 0.2 Hz, creating a pause of 5 s (post-rest beat). It is visible from the normalized traces in Figure 7B that the investigated trabecula displayed a negative force-frequency relationship (FFR), i.e., contraction and Ca^2+^ signal amplitudes were inversely related to pacing frequency. In the same experiment, 100 nM of the beta-1/2 receptor agonist isoprenaline was added to the perfusion buffer solution, which resulted in a nearly threefold increased contraction force and twofold increased Ca^2+^ signal. The FFR between 0.2 Hz and 1.5 Hz appeared less negative than under control conditions (Figure 7C), but showed a steeper negative slope at higher frequencies. Closer inspection of individual beats at different pacing rates revealed that the apparent difference in FFR at higher rates occurred because capture rate was increased in response to isoprenaline. While under control conditions only every second stimulus was captured starting at 2 Hz, each stimulus was captured after addition of isoprenaline (Figures 7D,E). Furthermore, when comparing the post-rest beats with the those recorded at 0.2 Hz, we observed that the relative increase of both contraction force and Ca^2+^ signal after the pause was higher under control conditions. This fits well to reports of a reduced post-rest response at high SERCA activity (Bluhm et al., 2000), because isoprenaline stimulates the SERCA. Figure 7 also shows that Ca^2+^ signals and contractility can be investigated simultaneously in cultivated trabeculae and that trabeculae respond as expected to beta-adrenergic stimulation. Of note, some trabeculae used for Ca^2+^ imaging were placed back on the cultivation system and could be successfully kept in culture for several days after the imaging. This suggests that the used Ca^2+^ indicator does not exert toxic effects and allows for repeated assessment of Ca^2+^ signals within the same trabecula.

### Effects of Beta-Adrenergic Stimulation on Contraction Parameters, Refractory Period, and Maximum Captured Frequency

**FIGURE 8:**
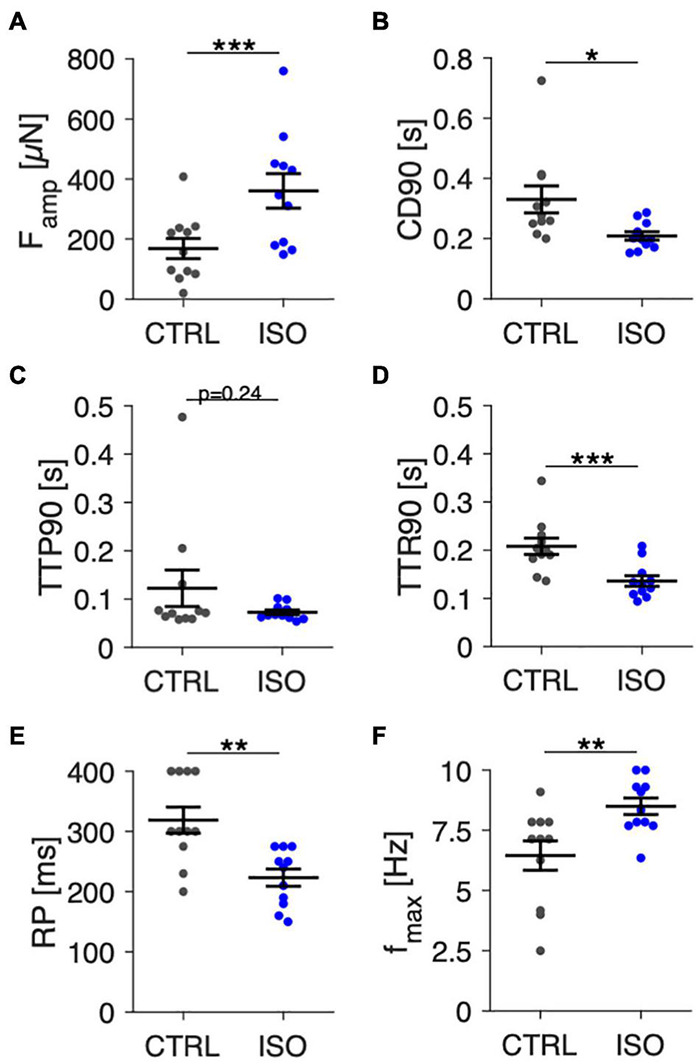
Effect of beta-adrenergic stimulation by isoprenaline on contraction amplitudes, kinetics, and pacing response in trabeculae at 7 days in culture. **(A)** Contraction force amplitudes (*F*_amp_) at control conditions (CTRL) and 5 min after addition of 100 nM isoprenaline (ISO), 1 Hz pacing. **(B)** Contraction duration (CD90), **(C)** time to peak (TTP90), and **(D)** time to relaxation (TTR90) at 1 Hz pacing. **(E)** Refractory period (RP), **(F)** Maximum frequency captured (*f*_max_). *N* = 11/3 trabeculae/samples. A paired *t*-test was applied. *p < 0.05, **p < 0.01, ***p < 0.001.

In Figure 8 we present data from 11 trabeculae that were treated with 100 nM isoprenaline at day 7 in culture. Contraction parameters, RP and *f*_max_ were evaluated directly before and 5 min after application of isoprenaline. Contractile force measured at 1 Hz (Figure 8A) increased by 114% from 168 ± 35 μN to 361 ± 60 μN (*p* < 0.001, paired *t*-test). CD90 decreased from 331 ± 47 ms to 209 ± 15 ms (*p* < 0.05), which predominantly resulted from a faster relaxation time (Figures 8B–D). RP decreased from 319 ± 22 ms to 223 ± 15 ms (Figure 8E), and *f*_max_ increased from 6.4 ± 0.6 Hz to 8.5 ± 0.4 Hz (Figure 8F), both being in accordance with shortened action potentials, increased excitability and accelerated mechanical response expected after beta-adrenergic stimulation. These results show that beta-adrenergic response is still present after 1 week in culture. They also let us conclude that the protocols used to determine RP and *f*_max_ are capable of detecting relevant differences.

### Tissue Microstructure

**FIGURE 9:**
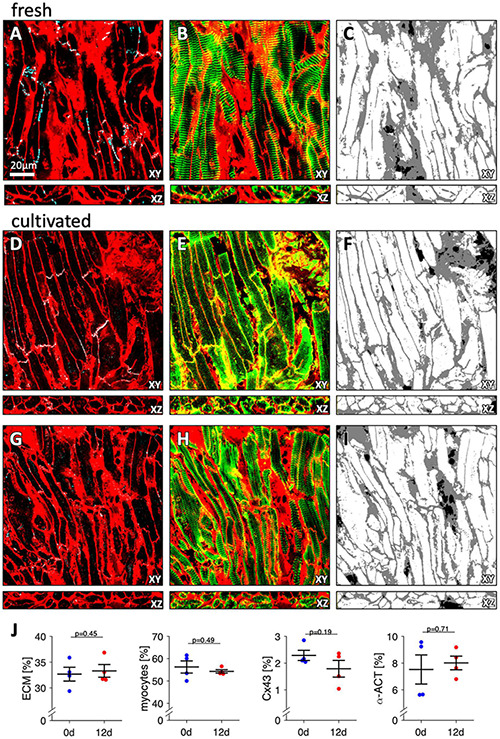
Three-dimensional microscopic images of fresh and cultured trabeculae stained with WGA for the extracellular matrix and with antibodies against Cx43 and alpha-actinin. **(A)** XY- and XZ-view of the WGA (red) and Cx43 signal (cyan) of a fresh (non-cultivated) trabecula. Note that the overlay of the signals appears white. The scale bar applies to all images. **(B)** XY- and XZ-view of the WGA (red) and alpha-actinin signal (green) of the same region shown in panel **(A)**. Note that the overlay of the signals appears yellow. **(C)** Extracellular matrix (gray) and myocytes (white) after computational segmentation and classification of the image stack shown in panels **(A,B)**. **(D–F)** Images of a trabecula cultured for 12 d presented in analogy to panels **(A–C)**. **(G–I)** Images of the same trabecula as in panels **(D–F)** from a different region, presented in analogy to panels **(A–C)**. **(J)** Volume fractions of the extracellular matrix (ECM), myocytes, Cx43, and alpha-actinin (α-ACT). The results from three image stacks of each sample were averaged and treated as one data point. Paired, two-sided *t*-test. *N* = 4 trabeculae from 4 specimens (patients).

To investigate effects of tissue culture on tissue and cardiomyocyte microstructure, we used immunofluorescent staining and confocal microscopy to compare four fresh trabeculae with four trabeculae from matching specimens (patients) after 12 d in culture (Figure 9). Fresh trabeculae displayed patches of fibrotic tissue and clearly visible Cx43 staining between cardiomyocyte borders (Figure 9A). Alpha-actinin (aACT) staining was visible in most cardiomyocytes and showed a regular sarcomere pattern (Figure 9B). The segmentation and classification of the imaged volume into cardiomyocytes and extracellular matrix (ECM) is presented in Figure 9C. Black regions indicate regions without any WGA or aACT signal, i.e., gaps in the tissue resulting from processing, cardiomyocytes without aACT staining, or other cell types, for example fibroblasts or endothelial cells. Figures 9D–I show two regions imaged in a trabecula from the same specimen (patient) that was cultivated for 12 d. In one region (Figures 9D–F), there was abundant Cx43 signal, similar to the fresh tissue and relatively weak but clearly visible aACT staining in most cardiomyocytes. The second region (Figures 9G–I) displayed only small clusters of Cx43, which were found not only at myocyte borders, but also intracellularly. The aACT signal intensity, however, was stronger. Quantitative analysis of the three-dimensional images indicated comparable amounts of ECM, myocytes, Cx43 and aACT in fresh and cultivated trabeculae (Figure 9J). This allows the conclusion that tissue culture preserved cardiomyocyte and tissue microstructure and did not cause additional fibrosis.

### Expression of Genes Related to Ca^2+^ Cycling and Electrophysiology

**FIGURE 10:**
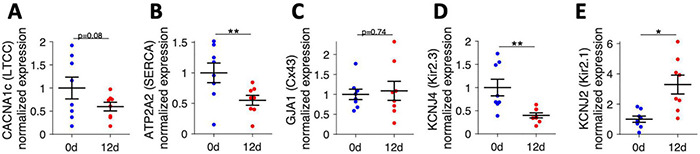
mRNA expression of genes related to Ca^2+^ cycling and electrophysiology analyzed by quantitative RT-PCR in fresh trabeculae (0 d) and trabeculae cultivated for 12 days (12 d). **(A)** CACNA1c subunit of the L-type Ca^2+^ channel (LTCC), **(B)** ATP2A2, encoding the sarcoplasmic/endoplasmic reticulum calcium ATPase 2 (SERCA2), **(C)** GJA1, encoding connexin-43 (Cx43), **(D)** KCNJ4, encoding the Kir2.3 subunit of the inwardly rectifying potassium channel, and **(E)** KCNJ2, encoding the Kir2.1 subunit of the inwardly rectifying potassium channel. *N* = 8/8 trabeculae/samples. A paired, two-sided *t*-test was applied. *p < 0.05, **p < 0.01.

Finally, we selected several genes that encode proteins important for cardiomyocyte Ca^2+^ cycling and electrophysiology and analyzed the mRNA expression of these genes by quantitative RT-PCR (Figure 10). After analyzing expression levels in 8 fresh trabeculae and 8 cultivated trabeculae from matched specimens (patients), we found that the CACNA1c subunit of the L-type Ca^2+^ channel had a trend toward lower expression after 12 d of culture, although statistical significance was not reached (*p* = 0.08). However, mRNA expression of ATP2A, the gene encoding the sarcoplasmic/endoplasmic reticulum calcium ATPase 2 (SERCA2) was significantly downregulated. GJA1, encoding Cx43, did not change significantly, but showed higher variance in cultivated trabeculae, which confirmed the results from confocal microscopic analysis of Cx43. We also investigated two subunits of the inwardly rectifying potassium channels that have been reported to be altered in atrial fibrillation (Deshmukh et al., 2015). The mRNA expression of KCNJ4, encoding Kir2.3, was downregulated by half, while KCNJ2, encoding Kir2.1, was about threefold upregulated. These data show that gene expression is altered in culture. They also demonstrate that it is possible to extract enough RNA from fresh and cultivated trabeculae to perform gene expression analysis with standard qPCR.

## Discussion

Due to non-negligible differences between human and animal cardiac physiology, testing hypotheses or verifying results on human cardiac cells and tissues is important. However, living human myocardium is difficult to obtain and may be even more difficult to use because the amount, quality, and variability of the specimens are often insufficient for conclusive experiments, especially those requiring preservation of cells or tissues over longer periods. As novel methods for stable long-term culture of human ventricular myocardium were recently presented, the question arose whether the principle of these techniques could be applied to human atrial tissue. Our results show that trabeculae (pectinate muscles) prepared from human right-atrial appendages can be maintained in culture in a commercially available device for up to 3 weeks under constant electrical stimulation with a frequency of 1 Hz, which corresponds to the human resting heart rate. We show that, although there is an initial transient decline in function, structure and function of trabeculae cultured for nearly 2 weeks are comparable to those of non-cultured (fresh) trabeculae. Furthermore, we describe a set of methods that can be used to acquire important functional parameters.

### Tissue Preparation and Viability in Culture

When comparing the presented method with that published for ventricular slices (Fischer et al., 2019), less time is needed for the preparation because the atrial tissue does not need to be cut into slices with a vibratome. Instead, trabeculae can be dissected with scissors and a scalpel. This makes the method not only faster, but also less expensive. Another advantage is that human atrial tissue can be obtained regularly during open heart surgery by removal of the RAA tip for insertion of the atrial cannula of the heart-lung machine (Boldt et al., 2004; Nummi et al., 2017). In contrast, sufficient amounts of ventricular tissue can be obtained only during implantation of ventricular assist device or after heart transplantation (Seidel et al., 2017a). We did not investigate if sliced atrial myocardium would be similarly or even better suited for tissue culture. However, in two pilot experiments where the wall of the RAA was sliced, we were not able to create slices of sufficient quality because of the irregular cardiomyocyte orientation and trabeculated structure. It could be investigated in future studies if the free wall of whole atria obtained, for instance, after a heart transplantation is better suited for slicing with a vibratome. The success rate in our study was close to 75%, that is 7–8 of 10 prepared trabeculae responded to electrical stimulation after at least 12 d in culture. This is in the range of success rates reported by other studies using human myocardium (Fischer et al., 2019; Fiegle et al., 2020). A distinct difference to ventricular slice culture was that we never observed initial tonic contracture, which has been reported to be a sign of tissue damage in human ventricular myocardium. It is possible that atrial tissue behaves differently when damaged or that hypercontracture did not occur because we had significantly shorter storage times than reported for ventricular tissue samples that were shipped on ice (Fischer et al., 2019).

### Contractility and Estimated Wall Stress

The changes in contractility observed over culture time (see Figure 4) resembled those reported for ventricular slices, with an initial decay and subsequent recovery. However, recovery occurred faster, because initial contraction amplitudes were regained after approximately 1 week, in contrast to 2–3 weeks reported for ventricular slices (Fischer et al., 2019). The decline and recovery of contractility could reflect adaptation of the tissue to culture conditions, including auxotonic contraction, a different extracellular milieu or cessation of catecholaminergic stimulation. A major difference in comparison to ventricular slices was visible when considering absolute contraction forces. Although some trabeculae produced forces higher than 1 mN (Figure 6A), most trabeculae reached contraction forces of 300–500 μN, which was about 10-fold smaller than in ventricular slices (Fischer et al., 2019; Abu-Khousa et al., 2020) and smaller than in other studies measuring isometric force in RAA trabeculae (Guo et al., 1983; Brixius et al., 1997). In the loaded state, most trabeculae exhibited a width of 1–2 mm and a height of 0.5–1 mm, resulting in an estimated cross-sectional area of 0.5–2 mm^2^. This yields a wall stress of about 0.15–1 mN/mm^2^, which is low when compared to the wall stress of up to 10mN/mm^2^ reported for isometric force measurements in human RAA trabeculae (Brixius et al., 1997). However, the mentioned studies (Guo et al., 1983; Brixius et al., 1997) measured isometric forces, which are commonly greater than forces developed during isotonic or auxotonic contraction as measured here. In fact, the ratio of wall stress during auxotonic contraction estimated here to isometric wall stress reported by other studies in RAA myocardium (≈1:10) is similar to the ratio of auxotonic wall stress measured in human ventricular slices [1–3 mN/mm^2^ (Fischer et al., 2019; Abu-Khousa et al., 2020)] to isometric wall stress in human ventricular muscle strips [10–20 mN/mm^2^ (Mulieri et al., 1992)]. Contraction duration CD90 found immediately after installation and after 12 d of culture (367 ± 50 and 414 ± 57 ms) and relative contributions of TTP90 (118 ± 16 and 155 ± 35 ms) were close to values found in other studies on RAA trabeculae [twitch time 433 ms and TTP 110 ms (Brixius et al., 1997)] and shorter than in human ventricular muscle strips [CD 634–747 ms and TTP 157–167 ms (Mulieri et al., 1992)].

### Assessment of Contraction Parameters, Refractory Period, and Maximum Captured Frequency

Our results show that the protocols and computational analyses used to assess contraction force, TTP90, TTR90, CD90, RP and *f*_max_ are sensitive enough to detect the changes expected after beta-adrenergic stimulation (see Figure 8). This finding also supports the conclusion from Figure 6 that trabecula function was similar at 0 d and after 12 d in culture, because no differences were found using the same analysis that detected differences after beta-adrenergic stimulation. However, while the interpretation of contraction amplitude and kinetic parameters TTP90, TTR90 and CD90 is straight-forward – they respectively correspond to actively developed force, contraction velocity, relaxation velocity and total duration of a contraction – it is more complicated to interpret RP and *f*_max_. The results shown in Figure 5 indicate that RP increases when an APD-prolonging drug (dofetilide) is applied (Tsujimae et al., 2007). In turn, the results in Figures 7, 8 indicate that isoprenaline, which has been shown to significantly decrease APD under steady-state conditions at normal to high extracellular Ca^2+^ (Munakata et al., 1982), decreases RP. Thus, the RP as assessed here seems to correlate with APD. This may also explain the data in Figure 7D, where at 3 Hz every other stimulus is captured although Ca^2+^ levels are already at baseline, suggesting that electrical excitability has not recovered yet, whereas after addition of isoprenaline, each stimulus is captured (Figure 7E). Still, it is important to note that the RP reported here cannot be set equal to the electrical refractory period or even APD, because a mechanical, not an electrical signal was measured. One should keep in mind that the threshold for considering an electrical stimulus as captured or non-captured was chosen somewhat arbitrarily. We considered each stimulus followed by a force increase larger than the noise level as captured. Another possibility would be to define a minimum amplitude that must be reached, for example 50% of the S1 beat. Such changes in definition would shift the refractory periods to larger intervals, but likely preserve the relative changes. Thus, it is important to define a fixed criterion as done here and apply this to all tissues investigated. The definition used here fits best to the absolute refractory period. Using a larger threshold, for instance 90% of the S1 beat, might be closer to the relative refractory period. Similar considerations apply to *f*_max_. The number of beats that had to be successively captured was arbitrarily set to 5, and, again, we considered very small beats as captured as long as they significantly exceeded the noise level. This may explain the relatively high values found for *f*_max_ (see Figures 6F, 8F). Modifications of the presented protocol for RP assessment could be smaller steps of the S2 beat at high S2 beat intervals. In our protocol, we used steps from 750 ms to 500 ms and then to 400 ms, which reduced the resolution of RP determination in trabeculae with high RP values (Figure 6E). To investigate how well the presented protocol works to detect the actual electrical refractory period and action potential frequency, one could design experiments with parallel voltage recording, for example using voltage sensitive dyes (Lang et al., 2011) or sharp electrodes (Peinkofer et al., 2017).

### Calcium Imaging

The Ca^2+^ indicator used here (Calbryte 520-AM) was recommended in other protocols describing Ca^2+^ imaging in human cardiac tissue (Borysova et al., 2021). According to the manufacturer it has a reported dissociation constant (K_D_) of 1200 nM. This would lead to a nearly linear relationship between the expected intracellular Ca^2+^ concentrations of 200–1000 nM (Backx et al., 1995) and the fluorescence signal. In Figure 7 the Ca^2+^ signal increased nearly twofold after addition of 100 nM of isoprenaline and also showed a clear increase during the post-rest beat. This is as expected from experimental and modeling studies reporting a 1.5- to 2-fold increase in intracellular Ca^2+^ after beta-adrenergic stimulation (Lyon et al., 2020) and suggests that the indicator was not saturated. Hence, relative changes in Ca^2+^ transient amplitude as well as Ca^2+^ transient kinetics can be studied with the presented approach. High-affinity Ca^2+^ indicators, for example Fluo-3 or Fluo-4, have been used frequently in preparations of intact hearts (Johnson et al., 1999; Kaneko et al., 2000). However, earlier studies suggest these dyes may saturate within the physiological range of intracellular Ca^2+^ (Stout and Reynolds, 1999; Kong and Fast, 2014). After Ca^2+^ imaging, we placed two trabeculae back into the incubator and were able to retain culture for several days without obvious negative effects. This would allow for repeated Ca^2+^ imaging in the same trabecula or slice. Although not investigated systematically here, other studies also reported low toxicity and repeated measurements with Calbryte (Liao et al., 2021). For several reasons, however, it is not reasonable to compare absolute values of the Ca^2+^ signal between different trabecula. First, it is unknown how the thickness of the tissue in the imaged region influences the signal. Second, variations in loading efficiency may have large effects on signal intensity. Future studies might use ratiometric Ca^2+^ indicators or combine Ca^2+^ indicators with additional dyes for voltage imaging.

### Tissue and Cardiomyocyte Microstructure

We used three-dimensional confocal microscopy to assess culture-induced changes in cardiomyocyte structure and tissue composition. Our results suggest that tissue culture preserved cardiomyocytes and did not cause fibrosis or heavy fibroblast proliferation, which can become problematic and may require pharmacological interventions in cardiomyocyte cultures (Boateng et al., 2003). It is possible that the intact tissue structure prevents fibroblast activation or that growth factors necessary for fibroblast activation are missing because serum-free culture medium was used. It would be interesting to investigate in future studies if and how fibrosis can be induced. Confocal data also indicate preserved gap junctions and sarcomeres, although some variations in staining efficiency of aACT and volume density of Cx43 were observed (Figure 9).

### Limitations

A limitation of the method is that the used tissues were obtained from patients suffering from cardiac diseases. Thus, although only patients with sinus rhythm were included, the results presented may differ in atrial trabeculae from healthy hearts. In some countries, however, healthy donor hearts are available for research through dedicated organizations.

During the first 2 weeks of culture, the function of trabeculae was not stable, but showed a transient decline and approached initial values after 12 days. This may reflect adaptation to culture conditions, which differ in several aspects from *in vivo* conditions, for example, hormonal stimulation, oxygen pressure, force-length-relationship during contraction or electrical stimulation. We observed mRNA downregulation of Ca^2+^ handling proteins, such as SERCA. This was also reported for ventricular slices (Fischer et al., 2019), where after 4 weeks in culture, gene expression partially returned to the non-cultured level. Our study suggests that this may apply also to human atrial tissue. Therefore, depending on the experimental setup and research question, for instance short-term or long-term drug effects, it can be appropriate to wait until stabilization of functional parameters before treatment or to treat immediately after installation in culture. In any case, time-matched controls should be used to account for culture-induced effects.

### Summary and Outlook

The method presented here describes an *in vitro* model of beating intact human atrial myocardium that can be readily implemented provided that RAA tissue is available. Because the atrial tissue is functionally and structurally stable for at least 3 weeks, we expect that the model can be used to study long-term drug and hormonal effects, mechanical and electrical stimuli to elucidate general atrial physiology and mechanisms of disease. It is also conceivable that the high stability will enable toxicity screening, drug discovery, co-culture with stem-cell derived cardiomyocytes, and gene delivery or silencing. These applications might be particularly useful to investigate the most common disease of the human atrium, which is atrial fibrillation.

## Data Availability Statement

The raw data supporting the conclusions of this article will be made available by the authors, without undue reservation.

## Ethics Statement

The studies involving human participants were reviewed and approved by Ethics Committee of the University of Erlangen-Nürnberg. The patients/participants provided their written informed consent to participate in this study.

## Author Contributions

MK, DF, and TS performed experiments, analyzed the data, and drafted the manuscript. CH and MK performed patient education, and acquired patient samples and patient data. TS, TV, CH, and MW designed the study. MK performed this work in (partial) fulfillment of the requirements for obtaining the degree “Dr. med.”. All authors critically revised the manuscript.

## Conflict of Interest

TS is shareholder of InVitroSys GmbH. The remaining authors declare that the research was conducted in the absence of any commercial or financial relationships that could be construed as a potential conflict of interest.

## Publisher’s Note

All claims expressed in this article are solely those of the authors and do not necessarily represent those of their affiliated organizations, or those of the publisher, the editors and the reviewers. Any product that may be evaluated in this article, or claim that may be made by its manufacturer, is not guaranteed or endorsed by the publisher.
