# Corrigendum: long-term cultivation of human atrial myocardium

**DOI:** 10.3389/fphys.2023.1206654

**Published:** 2023-05-03

**Authors:** Maximilian J. Klumm, Christian Heim, Dominik J. Fiegle, Michael Weyand, Tilmann Volk, Thomas Seidel

**Affiliations:** ^1^ Institute of Cellular and Molecular Physiology, Friedrich-Alexander-Universität Erlangen-Nürnberg, Erlangen, Germany; ^2^ Department of Cardiac Surgery, Friedrich-Alexander-Universität Erlangen-Nürnberg, Erlangen, Germany

**Keywords:** human atrium, calcium imaging, tissue culture, *in vitro*, confocal microscopy, gene expression, refractory period

In the published article, there was an error in **Author Contributions**. The following sentence was missing. MK performed this work in (partial) fulfillment of the requirements for obtaining the degree “Dr. med.”.

The authors apologize for this error and state that this does not change the scientific conclusions of the article in any way. The original article has been updated.

