# Supplementary material for: Long-Term Cultivation of Human Atrial Myocardium
Source: Front Physiol. 2022 Feb 23;13:839139. doi: 10.3389/fphys.2022.839139 (PMC8905341; doi:10.3389/fphys.2022.839139)
Supplement: Supplementary file 3 [file Data_Sheet_1.PDF]

Step-by-step protocol for culture of human atrial trabeculae

NOTE: To reduce the risk of infection, perform all steps marked with '**!**' under laminar flow

1 General Preparations

1.1 Media and buffers

! Prepare culture medium as listed in supplementary table
(2.5 ml per chamber, can be stored at 4˚ C for up to 1 week).
- Warm up culture medium and MTT buffer to 37° C and place it in the incubator with 5 % CO_2 atmosphere for pH equilibration.

1.2 Instruments and materials

! Disinfect two fine forceps, micro-scissors and a rubber pad with 2-propanol and let them air-dry.
! Fill two large (100mm), one medium (65 mm) and one small (30 mm) Petri dish with cold (4°C) storage solution and keep them on a cooled plate
! Disinfect plastic triangles with 2-propanol and transfer them to a small Petri dish filled with storage solution.
! Get tissue adhesive ready.
- Connect the cultivation system to the power supply and to the computer.
- Start the MyoDish Software and open the connection to the device.

1.3 Assembly of cultivation chambers

! Submerge cultivation chambers in 2-propanol for 5 min, then let them air-dry
! Connect sensor boards and cultivation chambers with two hex bolts
! Install graphite electrodes in the cultivation chamber with forceps and connect wire ends to the sensor board sockets
NOTE: Make sure there is enough distance between the electrodes and the spring wire (>1.5 mm)
! Fill each chamber with 2.4 ml cultivation medium and cover with a culture dish lid
- Place the chamber on the socket on the main plate of the cultivation system
- Start agitation of the culture system at 60 rpm
- Set pacing frequency to 0.5 Hz with 3 ms pulse duration (biphasic pulse) and 50 mA current.

2 Dissection and preparation of trabeculae

! Put rubber pad into a large Petri dish with cooled storage solution on cooling plate
! Transfer specimen of the right auricular appendage to the dish and inspect
! Incise the lateral border with scissors
! Open up the appendage and pin the sides to the rubber pad with sterile cannulas.
! Dissect trabeculae using micro-scissors and forceps. Do not overstretch the trabeculae.
! Transfer the dissected trabeculae to the medium-sized petri dish with storage solution and keep on ice / cooling plate.
! Transfer one trabecula to the inverted lid of a large Petri dish and cover with 200 µl storage solution.
! Drop approx. 50 µl of tissue adhesive onto a dry area of a Petri dish lid
! Dip the broad edge of a plastic triangle into the tissue adhesive and attach to one end of the trabecula with the edge perpendicular to the presumed fiber orientation.
! Repeat accordingly for the other end of the trabecula.
! Store prepared trabeculae in medium petri dish with cold storage solution on cooling plate

3 Installation of trabeculae into tissue culture

- ! Disinfect a heating mat (37-39° C) with ethanol and place.
- ! Take one chamber at a time out of the incubator and place it on the heating mat.
- ! Adjust distance of wires with a hex key to about 1 cm for easier mounting
- ! Remove the lid of the chamber. Take one prepared trabecula with a curved forceps and mount the plastic triangles on the posts of the spring wire and the adjusting wire.
  - o NOTE: Grab the plastic triangles rather than the tissue, do not stretch the trabeculae.
- ! Adjust the distance of the wires with a hex key according to the trabeculae length, but do not stretch the tissue.
- ! Cover the cultivation chamber with the lid
- Stop rocking of the cultivation system main plate, place chamber back to its socket and then resume rocking for medium agitation.
- Using a hex key turn the screw for preload adjustment while watching the monitor. First, turn counter-clockwise to verify that no preload is present (diastolic value should not change). Then, turn clockwise until the diastolic preload reaches the desired value.

4 Exchange of culture medium

NOTE: 1.6 ml of the culture medium should be exchanged every 24 hours or 72 hours, depending on the tissue and culture conditions.
NOTE: For stable culture, it is important to avoid temperature changes. Make sure to always keep tissues at 37° C. Never add culture medium cooler than 36° C.

- Pre-warm the culture medium to 37° C and equilibrate at 5 % CO_2 and 37° C.
- ! Disinfect the heating mat and heating block with ethanol, place it in the laminar flow workbench and set to approx. 38° C.
- ! Transfer the tube containing the culture medium to the heating block.
- ! Take out the cultivation chamber and place it on the heating mat.
- ! Remove the chamber lid, aspirate 0.8 ml (24 h exchange interval) or 1.6 ml (48-72 h exchange interval) of the medium and discard.
- ! Add 0.9 ml (24 h exchange interval) or 1.8 ml (48 h exchange interval) fresh medium.
  NOTE: Depending on relative humidity, evaporation may be smaller or greater.
  NOTE: Do not directly pour the medium over the trabeculae to avoid mechanical stress.
  NOTE: If culture medium is spilled on the edges of the chamber or the sensor board, use a sterile tissue or swab to carefully remove the medium.
- ! Cover the chamber with the lid and place it back to its socket on the setup.

MTT Viability Assay

- Take the respective chamber from the setup and place on a heating mat at 37° C.
- Remove the stimulation electrodes using forceps.
- Aspirate and discard the culture medium and wash with 1 ml pre-warmed MTT buffer.
- Aspirate and discard MTT buffer from chamber and add 2 ml MTT buffer supplemented with 0.5 mg/ml tetrazolium dye.
- Incubate for 20 min with agitation (60rpm) at 37° C and 5% CO_2.
- Remove the dye solution and wash 2x with 1 ml MTT buffer.
- Unmount the trabeculae from the cultivation chamber, remove the plastic triangles with a scalpel or scissors and snap-freeze the trabecula dry at -80° C.
- Dissolve formazan dye from tissue in 1 ml DMSO for 30min at 37° C and 60rpm agitation. Detect absorbance at 555 nm in a photometer.
- Use the remaining tissue to perform a total protein analysis (e.g., Bradford assay) for normalization of formazan absorbance.
