# Supplementary material for: Long-Term Cultivation of Human Atrial Myocardium
Source: Front Physiol. 2022 Feb 23;13:839139. doi: 10.3389/fphys.2022.839139 (PMC8905341; doi:10.3389/fphys.2022.839139)
Supplement: Supplementary file 4 [file Data_Sheet_2.PDF]

# – Supplemental Material –

Long-term cultivation of human atrial myocardium

M. J. Klumm[1,2†], C. Heim[2†], D. J. Fiegle[1], M.Weyand[2], T. Volk[1], T. Seidel[1]

1 Institute of Cellular and Molecular Physiology, Friedrich-Alexander-Universität Erlangen-Nürnberg, Erlangen

2 Department of Cardiac Surgery, Friedrich-Alexander-Universität Erlangen-Nürnberg, Erlangen, Germany

1 Materials and Equipment

1.1 Buffers and Media

Name	Solvent	Composition / Supplements	Concentration
Storage solution	ddH_2O	NaCl	138 mmol/l
		NaH_2PO_4	0.33 mmol/l
		butadiene-monoxide (BDM)	30 mmol/l
		Glucose	10 mmol/l
		HEPES	10 mmol/l
		KCl	5.4 mmol/l
		$CaCl_2$	0.5 mmol/l
MTT buffer	ddH_2O	$CaCl_2$	1.8 mmol/l
		butadiene-monoxide (BDM)	30 mmol/l
		Glucose	10 mmol/l
		HEPES	10 mmol/l
		KCl	5.4 mmol/l
		$MgCl_2$	1 mmol/l
		NaCl	136 mmol/l
		NaH_2PO_4	0.33 mmol/l
Ca^{2+} imaging buffer	ddH_2O	NaCl	138 mmol/l
		KCl	4 mmol/l
		$CaCl_2$	2 mmol/l
		$MgCl_2$	1 mmol/l
		NaH_2PO_4	0.33 mmol/l
		HEPES	10 mmol/l
		glucose	10 mmol/l
Culture medium	M199 (Sigma, M4530)	Penicillin	10 U/ml
		Streptomycin	10 µg/ml
		Insulin	10 µg/ml
		Transferrin	5.5 µg/ml
		Selenium	6.7 ng/ml
		2-Mercaptoethanol	50 µmol/l
		Cortisol	20 nmol/l

1.2 Chemicals and reagents

**Name**	**Manufacturer**	**REF number**
4-(2-hydroxyethyl)-1-piperazineethanesulfonic acid (HEPES)	Roth	9105.3
Bovine serum albumine fraction V (BSA)	Roth	163.2
Butanedione monoxime (BDM)	AlfaAesar	114339
Bradford protein assay reagent	BioRad	5000006
Calcium chloride	Roth	5239.2
Cortisol	Sigma	H4001
Dofetilide	Sigma	PZ0016
Glucose	Merck	50997
Isoprenaline	Sigma	I5627
Magnesium chloride	Roth	A537.1
Normal goat serum	Sigma Aldrich	G9023
Paraformaldehyde	Sigma Aldrich	158127
Polyethylene glycol p-(1,1,3,3-tetramethylbutyl)-phenyl ether (Triton X-100)	Roth	3051.3
Potassium hydroxide	Merck	105032
Sodium chloride	Roth	9265.1
Sodium dihydrogenphosphate	Sigma Aldrich	S5011
Thiazolyl blue tetrazolium bromide (MTT)	Sigma	M2128
Penicillin/Streptomycin	Biochrom	A2213
Insulin Transferrin Selenium (100x)	Gibco	41400045
Pluronic Acid F127	Biotium	59005

1.3 Antibodies, fluorophores and dyes

Antibody/Reagent	Manufacturer	REF number	Dilution / concentration
Mouse anti α-actinin IgG1	Sigma	A7811	1:200
Rabbit anti connexin-43	Sigma	C6219	1:400
Goat anti-mouse IgG1-AlexaFluor 488	Invitrogen	A21121	1:200
Goat-anti-rabbit IgG-AlexaFluor 555	Invitrogen	A21428	1:400
4′,6-diamidino-2-phenylindole (DAPI)	Roth	63351	2 µg/ml
Wheat germ agglutinin (WGA) - AlexaFluor 647	Invitrogen	W32466	40µg/ml
Calbryte 520-AM Ca^{2+} indicator	AAT Bioquest	20651	10 µmol/l

1.4 PCR primers

Target gene (protein)	Fwd (5’-3’)	Rev (5’-3’)
CACNA1C(LTCC)	TGTCCGGAGTCCCAAGTCTC	CATGGCCTTGATGATGGAA
ATPA2 (SERCA)	ACAATGGCGCTCTCTGTTCT	ATCCTCAGCAAGGACTGGTTT
GJA1 (Cx43)	TACCAAACAGCAGCGGAGTT	TGGGCACCACTCTTTTGCTT
KCNJ2 (Kir2.1)	CTTACATGCCTCTGTACCCCC	TCTCTGGGAGCCTTGTGGTT
KCNJ4 (Kir2.3)	CTCTCGTCGGACCCTCC	GTGTCCGTGCATGTCCTGAA
EEF2	GGATGGGACACCCAACACTT	CTTTCTGGGGCAAAAGCCAC
HPRT1	GGCAGTATAATCCAAAGATGGTC	TTCAAATCCAACAAAGTCTGGC

1.5 Devices, tools and other materials

**Device/tool/material**	**Model**	**Manufacturer (REF)**
Confocal microscope	LSM780	Zeiss
Cryotome	CM3050S	Leica Biosystems
Culture Chambers	MyoDish Culture chamber, spring stiffness #2	InVitroSys
Embedding compound	O.C.T. Tissue Tek	Sakura (4583)
Fluorescence microplate reader	GENios	Tecan
Incubator	ICO240	Memmert
Mounting Media	Fluoromount G Sigma	Sigma (F4680)
Real-Time Thermocycler	StepOnePlus	Applied Biosystems
Tissue Adhesive	Histoacryl glue	Surgibond (ZGS)
Tissue Culture System	MyoDish 1	InVitroSys
Tissue homogenizer	T10 basic	IKA (0003737000)
Spectrophotometer	DS 11+	Denovix (31DS-11PLUS-B)
RNA isolation kit	NucleoSpin RNA-Kit	Macherey-Nagel (740955.50)
Reverse transcriptase kit	QuantiTect RT-Kit	Qiagen (205311)
PCR reagent	SYBR^{TM} Select assay	Thermo Fisher (4472903)
Inverted microscope	DM IRB	Leica (090134010000)
Fluorescence imaging system	FSI800, MyoCamS3, PMT400	IonOptix

2 **Supplemental Figure 1**

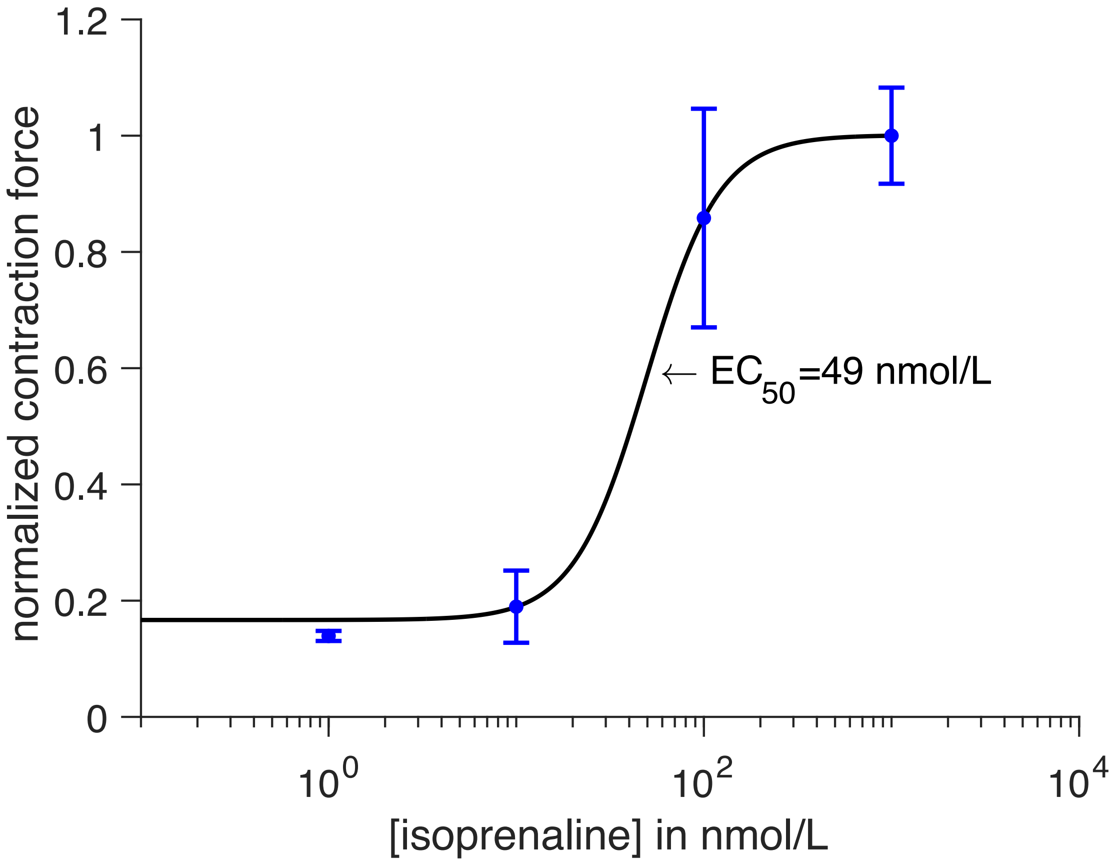

Supplemental Figure 1. Isoprenaline dose-response curve from 3 trabeculae immediately after installation in culture.
